# Cell-f identity of biomedical students: from energetic “mitochondrials” to gastronomic “lysosomics”

**DOI:** 10.3389/fmed.2026.1690470

**Published:** 2026-01-23

**Authors:** Teun J. de Vries

**Affiliations:** 1 Department of Periodontology, Academic Centre for Dentistry Amsterdam (ACTA), University of Amsterdam and Vrije Universiteit, Amsterdam, Netherlands; 2 Amsterdam University College, University of Amsterdam and Vrije Universiteit, Amsterdam, Netherlands

**Keywords:** bachelor education, biomedical education, honors college students, mitochondrium, physical activity, self-identity, sense of belonging

## Abstract

At the start of a Molecular Cell Biology course, 66 students from the biomedical track introduced themselves by identifying with a specific cell component. On the final exam, they were asked once again to name their favorite cell component at that moment—this time providing concrete details based on what they had learned throughout the course. Remarkably, the students named no fewer than 21 distinct components. Popular choices included mitochondria (20 students), the cytoskeleton (7), and the cytoplasm and ribosome (5 each), while more unusual responses featured the flagellum and GPCR receptors. Although the question called for a scientific explanation, only 20 students provided purely scientific answers. A total of 11 students responded with purely associative descriptions, without linking their choice to biological function. The remaining 35 students offered hybrid responses, blending newly acquired cell biology knowledge with personal reflections. The students connected cellular features to broader themes such as personality, personal growth, adaptability, relationship maintenance, organizational skills, hobbies such as physical exercise, and gastronomy. These findings show that cellular features and functions evoke a wide range of associations with aspects that are important in the lives of undergraduate students.

## Introduction

Courses related to Molecular Cell Biology play a central role in biomedical education. They teach the fundamentals of life at the DNA level and explore how cells respond to genetic alterations. Biomedical researchers aiming to improve clinical outcomes now have new opportunities. Advances in cell biology will help researchers make crucial contributions to patient care. The mapping of approximately 25,000 human genes, along with the identification of disease-causing mutations, holds great promise for the development of targeted treatments (1). Furthermore, with the advent of CRISPR-Cas9 technology, it is now possible to envision gene-editing approaches to correct undesired mutations (2).

Designing a modern Molecular Cell Biology course for undergraduate students pursuing biomedical or medical careers should go beyond traditional textbooks. While established resources such as Essential Cell Biology (3) provide a solid foundation, courses can be enriched by linking each topic to relevant genetic diseases (4). Given the abstract nature of the material, visual aids such as cartoons and animations are valuable tools for enhancing comprehension (5). Students should also be equipped to navigate the biomedical literature, which requires a basic understanding of the techniques used to detect nucleic acids and proteins (6, 7).

When Molecular Cell Biology is taught intensively in small groups—such as in honors colleges—fostering a sense of community is essential (8). One effective method is the elevator pitch approach (9), which is an icebreaker activity where students introduce themselves in under 2 min. In my course, I adapted this approach by asking students to identify a cell component they relate to and explain why. In addition to revealing their preferences, this exercise provided a global impression of the group’s overall level of knowledge. This is especially important when students are recruited from all over the world and have widely varying backgrounds in biological knowledge. To give the course a clear beginning and end, the final question of the final exam referred back to this elevator pitch moment. Students were asked about their favorite cell component at the end of the course, requiring them to integrate the knowledge they had gained throughout the course. The intention was to use the icebreaker activity as a means of assessing what the students had learned during the course, linking it to a favorite cell component. In this way, it provides insights into whether students are able to articulate in a specific way what they have learned during the course. By linking the choice of an organelle or other cell part, the teacher could gain insights into what really fascinated the students. The question was intended to be answered in a purely scientific manner. Surprisingly, many responses included personal reflections—touching on hobbies, friendships, and organizational skills. These outcomes, described in this article, suggest that cell biology naturally invites associations that extend beyond the academic content.

## Methods

### Description of the students and the course

Amsterdam University College (AUC) is an international liberal arts and sciences honors college, where all courses are taught in English. Admission is selective, based on academic performance and student motivation. AUC attracts students from across the globe, creating a vibrant and diverse learning environment. One of AUC’s distinctive features is its small class sizes—each course is limited to a maximum of 25 students, fostering close interaction and personalized instruction. Among the popular courses is Molecular Cell Biology, which is frequently selected by students pursuing biomedical or science tracks. In fact, a significant portion of the student body—likely more than half—envisions a future as either a medical doctor or a biomedical scientist. Another hallmark of AUC’s academic approach is its commitment to continuous assessment. The Molecular Cell Biology course, which runs from September to December, includes five graded components:

- Three exams, each contributing 22% to the final grade,
- A presentation on a selected topic (14%),
- An essay on the same topic (20%),

The presentation and essay are completed in pairs, encouraging collaboration and deeper engagement with the subject matter.

The course follows the framework of the widely used textbook “Essential Cell Biology” (ref) but also invests in a learning goal to make students competent and confident readers of cell biological literature, a skill assessed through the presentation and essay assignments. To achieve this, a flipped classroom approach was implemented, in which students engage with research methods for protein detection (ELISA, Western blotting, immunohistochemistry, and flow cytometry) (7). Building on insights gained during the COVID-19 period of online teaching (10), we invite inspirational guest lecturers to explain how the microbiome influences human health and behavior (11). A lecture on live cell imaging was delivered online by Sarah Dallas (United States) via Zoom (12). No fewer than 66 students enrolled in the 2024–2025 course, indicating that the course had to be taught in three groups.

### Exam question

The bonus question of the final exam was as follows:

“During the get-to-know-each-other during our first class, you have chosen a cell part (organelle, cytoplasm, membrane) that you identify with. Now, after the course give a short argument for your present choice and use fresh knowledge/terms that you have learned during Molecular Cell Biology. [sic].

Cell part I chose is …………………………………………. because ….”.

### Analysis and ethical issues

During the grading of the question, the students’ responses proved to be unexpectedly profound, offering insights into their analytical depth and originality. The answers were compiled into a table, and the idea arose to share their lucid responses (this article). Amsterdam University College does not have an Institutional Review Board; therefore, the following steps were undertaken. Since this was not—and could not have been—planned beforehand, I reached out to students via the digital teaching and learning platform Canvas to discuss the idea of publishing their answers. Anonymity was guaranteed, and any students who objected could notify me so that their answers could be excluded from the analysis. For students who no longer remembered their answers, I offered to provide them individually upon request. Importantly, students were given the option to opt out. To further guarantee that nothing in the article could harm the students or the institution, the pre-final version of the manuscript was read to the Head of Education and the Head of Sciences at Amsterdam University College, both of whom endorsed the publication of the findings. They provided written, signed consent for the publication of this article.

## Results

### Quantitative analysis

#### Students selected 21 different cell components, but mitochondria were the most popular

**Figure 1:**
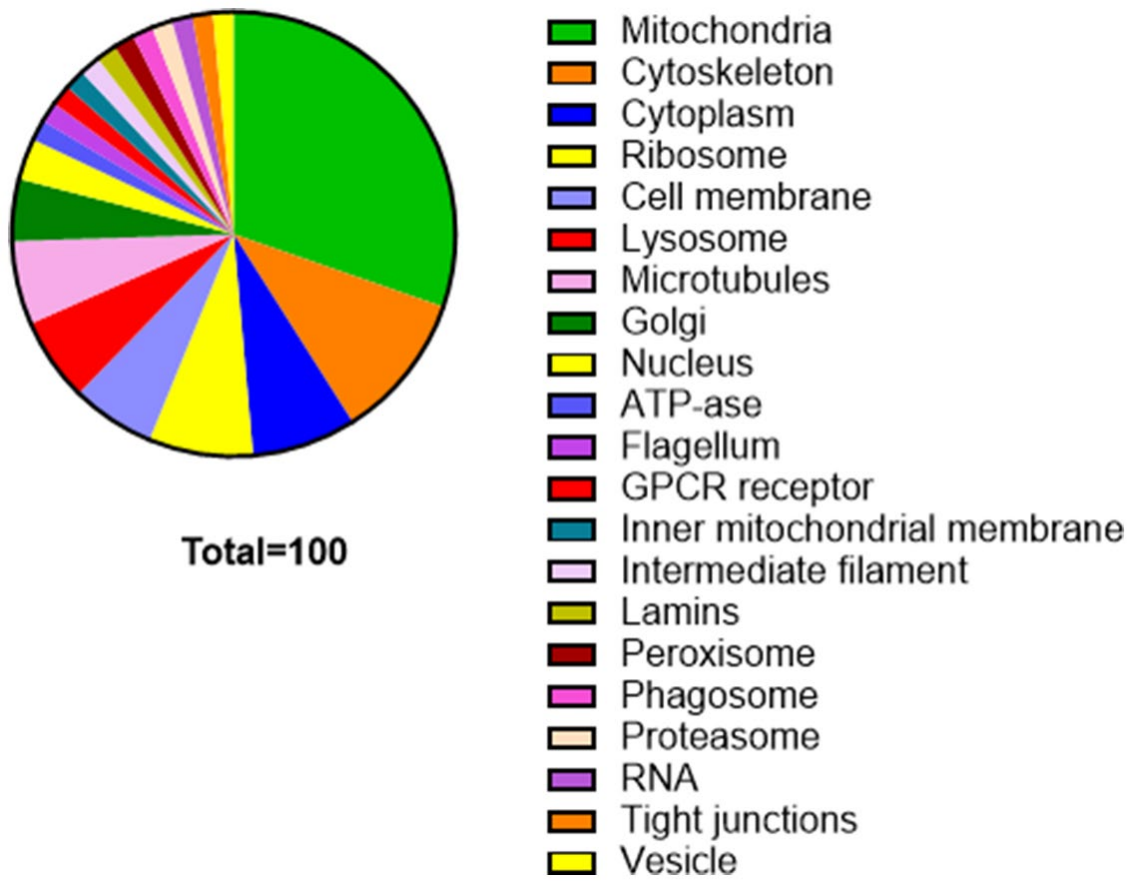
Cell components selected by the students. Percentages are shown. The main choice was mitochondria, followed by the cytoskeleton and others. Data are based on responses from the 66 students and are presented as percentages.

The frequency of cell components mentioned by the students is shown in Figure 1. All 66 students responded. No fewer than 21 cell components were mentioned. Mitochondria was mentioned by nearly one-third of the students (20), the cytoskeleton was mentioned by seven, and the cytoplasm and ribosome was mentioned by five. The cell membrane, lysosome, and microtubules were each mentioned four times. Some of the answers were more detailed and could also be assigned to broader components—for example, the inner mitochondrial membrane was categorized under mitochondria and the intermediate filament under the cytoskeleton. Other answers were even more specific, identifying individual proteins (such as GPCRs and lamins). The cytoskeleton, or components of the cytoskeleton such as microtubules, was never mentioned during the introduce-yourself session in the first class, indicating that the students’ knowledge of the cytoskeleton was acquired during this course.

#### Students responded with scientific answers, personal character traits, or hybrid responses

The way the question was phrased, beginning with “During the get-to-know-each-other during our first class, you have chosen a cell part …,” likely encouraged the students to incorporate personal characteristics into their answers. The answers could be categorized as purely scientific (1), focused on a character trait (3), or hybrid, in which the students eloquently combined learned cell biology concepts with personal traits (2) (See Table 1).

**Table 1:** Cell-f identification of 66 students in molecular cell biology.

Cell component, Student id	Answer	Cat.*
**Mitochondria (20)** 1	**I store knowledge in my brain** as potential (like electrochemical in mitochondria) and **release it all through my pen** (ATP synthase) **which creates my marks** (ATP)	2
2	**Because I also have two layers (aka personalities). One that a lot of people see (outer membrane is permeable for various small molecules) and one that only certain people can see (those who pay for tickets in the theater). Inner membrane is very selectively permeable (protons).**	2
3	The mitochondria as ATP synthase absolutely blows my mind. I have learned about the intrinsic DNA it has, the ability to fuse and do fission, nearly constantly to regulate itself. It just fascinates me, so inherent in the evolution of life in different biological kingdoms.	1
4	It harvests up to 50% of the energy stored in food and releases the rest as heat and **I am rarely ever cold**.	2
5	It provides the perfect microenvironment for ATP synthesis, with and elegant mechanism of using an electrochemical H + gradient fueled by e- transport within the mitochondria. The whole process is so effective that it produces 30 ATP molecules, as compared to just 2 in glycolysis.	1
6	**I bring an uplifting energy to any room I’m in.**	3
7	**The large caffeine amounts I consume give me lots of energy as ATP synthase creates in the mitochondrion using a proton gradient.**	3
8	Mitochondria are resilient organelles that can adapt under stress and, apart from being the powerhouse of the cell (which everyone knows), they also protect the cell through apoptosis and mitophagy, very assertive!	1
9	It is not only the powerhouse of the cell, but energy provider for the organism. Its process is super complicated to understand, like me. From protons, electrons, citric acid cycle and ATP synthesis and in order for mitochondria to work everything has to work sufficiently but the start is with food molecules from the cytosol, which **resonates with me because I am always hungry**.	2
10	Strategically uses resources to power (provide energy) for the rest of the processes.	1
11	It generates energy by the buildup of a lot of lovely protons (**friendly family**).	3
12	It’s product of ATP is essential to almost every cell function. Furthermore the cell’s ability to regenerate energy carrying molecules like NADH and FADH2 to use for glycolysis and the citric cycle is truly fascinating.	1
13	They are more complex than people think they are. You are taught from a young age that they generate energy but since taking this class I’ve seen how many functions they have in ATP production, fat metabolism and ROS. **They have multiple sides and layers to them just like every person.**	2
14	**I keep myself running and motivated, but I have a very chaotic structure and I’m constantly changing (referring to mitochondria as a web of constantly splitting and moving.**	3
15	It is involved in other aspects such as fat metabolism and Ca2 + storage. In many ways, the mitochondria **is an organelle aspire to be me: an intricate system which although overly complicated, manages to do things efficiently. I can at times rush into projects, but I always try to reach smaller goals one at a time and believe in the importance of taking your time and going step by step.** Clearly it is working for the mitochondria	2
16	**I am adaptable**, just like how mitochondria have antioxidant enzymes to protect against oxidative stress from ROS.	2
17	**I love to do sports and be active**, which requires a lot of ATP to drive the actin-myosin cross-bridge cycle in muscle cells. The ATP is generated in the mitochondria during the electron transport chain	2
18	**Maybe I would be able to produce the same energy to sustain life despite the unexpected changes in life through different compartments**	3
19	The mitochondria converts glucose and fatty acids into ATP. **I feel like I am personally a very energetic person, and I love to eat glucose-rich food**	2
20	It metabolizes fatty acids and **I’m excited to eat lots of fatty foods on Christmas day**	2
**Cytoskeleton (7)** 21	It provides support to the cell (**my community**) and helps transport molecules (**I like helping and interacting with others**).	2
22	It has a multi-layered system which makes it flexible, but still rigid, and effective at its job. The actin filaments while seemingly weak and easily broken, gives the cells much support and an overall structure, **also allows for the cell to go to places. I think out of the three that is the one I identify with.**	2
23	The cytoskeleton not only provides the cell with rigidity (intermediate filament), it is also crucial for cell movement (actin) and intracellular transport (microtubules), **which reflects how dynamic I am as a person.**	2
24	They are the real supporters and organizers of a cell. And **I believe my best trait is my organizational skill.**	2
25	I did not really know what it was before, but I have now learned that it encompasses 3 different types and structures (actin filaments, microtubules, and intermediate filaments), which are essential to the cell. They allow the cell to do many things, such as moving, crawling, dividing, contracting. It is just very interesting to see how, for instance, during cell division, dynamic instability increases because of M-Cdk and this is what allows the microtubules to look around for the kinetochines randomly, but at a faster speed.	1
26	**I give good advice in arguments or disagreements - this advice that holds friendships and relationships together is like the rope-like structure** of intermediate filaments that give the cell great tensile strength!	2
27	Though often lying under the radar, the cytoskeleton is a uniter of forces. **I resonate closely with this- the cytoskeleton’s multifaceted and ever adapting role** in enabling some of the cell’s most crucial functions from cell division to cell movement.	2
**Cytoplasm (5)** 28	Because it has a strong support system around it. Certain structures such as intracellular filaments, microfilaments, and actin filaments provide organizational structure to it strengthen its membrane, and offer dynamic features and opportunity. **I am similar to the cytoplasm since my support system (my family of protein filaments) are made up of my amazing friends that provide me with strength, my family that provides me a solid and healthy organizational structure, and last but certainly not least, my fabulous kind hearted professors who give me dynamic opportunity to expand my knowledge in line with my imagined career.**	2
29	It is actually filled with intermediate filaments, actin filaments, and microtubules and is not just a gel-like space with randomly positioned organelles	1
30	It’s much more complex than I thought! It houses tons of inhabitants by having a powerful, yet flexible skeleton, due to intermediate filaments, microtubules and microfilaments.	1
31	It adds shape and character to the larger whole (cell), it’s a part of, due to actin filaments.	1
32	**Is the land of opportunities. Where proteins are synthesized and dreams are made.**	3
**Ribosome (5)** 33	I can identify with them because of their location. They can be found free in the cytoplasm (**like me around the city**), but they are highly concentrated in the rough ER (**like me in science park- where I live and study) and spend most of my time; you will find “more” of me here than freely around the city.**	2
34	I identified as a ribosome because I am generally **the type of person who likes ensuring everything organized and functions smoothly and efficiently.** During the course, I learned that indeed ribosomes work efficiently to ensure proteins are assembled quickly and accurately, with mechanisms to check for errors during translation.	3
35	**Since I like cooking** I’d be a ribosome. Ribosome is formed by subunits and assembles at the methionine codon and many of them form an array of chefs, the polyribosome	1
36	**I am not a native speaker of English, and I need to translate information and sometimes also make mistakes (mutations).**	2
37	It is the site at which all the wonderful proteins are translated	1
**Cell membrane (4)** 38	**I protect and wrap around distinctive characteristics parts of me (organelles), which contribute differently but as a whole, very successfully to how I behave and function.**	3
39	**I connect well with others** as the proteins (integrins) in the membrane can connect with its surroundings and desmosomes and intermediate filaments can connect adjacent cells.	2
40	It’s a vital and sometimes forgotten about part of the cell. It’ sometimes hard to spot, but actually crucial in man y processes. It’s where desmosomes bind, hold intermediate filaments, keeping the cell intact. I also like the different junctions it takes part in, like tight junctions keeping the apical part and basal parts separate. It may seem insignificant, but when you know more about it, it’s so interesting and complex.	1
41	The plasma membrane consists of constantly shifting transmembrane proteins and channels. It uses intracellular resources (like ATP) to create membrane potential to accomplish tasks.	1
**Lysosome (4)** 42	The lysosome is very useful to digest and degrade things through its very enzymatic full interior.	1
43	It can form a phagolysosome to degrade the proteins of the pathogen. **Since I digest food easily, I resemble it.**	2
44	Lysosome is responsible for cleaning the waste products, forms a phagolysosome, and **I like cleaning**.	2
45	**I like eating!** Lysosomes are typically used for the breakdown (hydrolysis_ of molecules. They’re transported around the cell by microtubules to destination **(I bike around Amsterdam to get to nice restaurants)**	2
**Microtubules (4)** 46	Fascinating intracellular highways, the way kinesins and dyneins transport cell material along axons using ATP hydrolysis makes the complexity of biology so incredible (especially because of my interest in neuroscience)	1
47	**Academically, I feel that I thrive when the material is getting pilled on fast** and is interesting similar to a GTP-cap making the microtubules more stable but when the material is being put on too slow like tubulin **I get a chance to slip, I end up falling apart like a microtubule**.	2
48	**They are the “railroad” of the cell and help stuff move around, which I feel like I do every day. They also act like the logistic manager and move in the cell, which again is similar to me.**	2
49	I chose the microtubules because **I feel the same as them in the sense that if the conditions are not great (low temperature in the dorms) than my structure (my mood, efficiency) is disrupted and I cannot keep my integrity (I am in chaos); therefore, many processes are also disrupted (just like in my life).**	2
**Golgi (3)** 50	**I think a lot** so I will now just pick kinesin and dynesin for the purpose of like in to **walk a clear path and “transporting cargo” to my friends in need**.	2
51	**I like organizing things like to-do lists, schedules, and my room. But after the course, I also think that I like to help people, and without the Golgi apparatus helping the transport of molecules, the cell would not function.**	3
52	Because it helps vesicles containing proteins that need to be secreted to extracellular space, **just like I am trying to help young students leave their country**	2
**Nucleus (2)** 53	It is supported by nuclear lamins, a type of intermediate filaments	1
54	**It looks safe.** It is protected by the intermediate filament. The nucleus also looks cool as it can divide in two during mitosis	2
**ATPase** 55	**I need constantly a flow of hydrogen to stay productive. I like to generate ATP for others.**	2
**Flagellum** 56	I learned more about the flagellum, about the role of microtubules and dynamics in movement of flagellum which is very nice.	1
**GPCR receptor** 57	The GPCR receptor passes the membrane seven times and has three subunits. **My birthday is on 7th of March, so I identify with it.**	2
**Inner mitochondrial membrane** 58	ATP is produced here and provides energy for the body.	1
**Intermediate filament** 59	I can relate to its position when for example, the keratin in epithelial cells is resisting the external compressive pressure to maintain the cell shape.	1
**Lamins** 60	While supporting the nucleus’ strength and integrity in mitosis, they are forced to disassemble and reassemble. **My confidence is easily overcome by uncertainty.**	2
**Peroxisome** 61	They get rid of things toxic to the cell (i.e., reactive oxygen species).	1
**Phagosome** 62	**I love food and phagosomes form when the cells endocytose bacteria and degrade molecules to digest them.**	2
**Proteasome** 63	**I’m very good at seeing my own mistakes.**	3
**RNA** 64	It can take on many different roles in the cell and comes in many forms, mRNA, tRNA, microRNA… to name a few. It enables not only transcription of genes and translation to proteins but also I learned in this class the inhibition of expression of certain genes through microRNA and **I believe that it is additionally very fitting to my character because I sometimes micromanage to avoid negative impacts on mine or other’s lives.**	2
**Tight junctions** 65	**I get attached to my friends** (those would be the epithelial cells) and **I do not let anything go** (and this would be larger molecules)	3
**Vesicle** 66	They are always changing and adopting. They put on their coats (**new knowledge**) and go into the world carrying various things and come back with stories (new material) to bring home (the cell).	2

Bold text indicates parts of the answer that referred to personal traits rather than the scientific content. Cat. *Three categories were scored: (1) Purely scientific answers, (2) hybrid answers that combined both scientific and personal traits, and (3) purely personal traits.

The first category was selected by 21 students, almost one-third of the class, and was the intended answer. A highly analytical answer from one student (student 40 in Table 1), who chose the cell membrane, incorporated many other concepts learned during the course: “*It’s a vital and sometimes forgotten about part of the cell. It’s sometimes hard to spot, but actually crucial in many processes. It’s where desmosomes bind, hold intermediate filaments, keeping the cell intact. I also like the different junctions it takes part in, like tight junctions keeping the apical part and basal parts separate. It may seem insignificant, but when you know more about it, it’s so interesting and complex.” [sic].*

The second category, consisting of hybrid answers that combined concepts learned in the course with individual traits, was selected by more than half of the students (35 of 66). An example from this category, provided by a student who identified with the ribosome (student 35), is as follows: “*Since I like cooking I’d be a ribosome. Ribosome is formed by subunits and assembles at the methionine codon, and many of them form an array of chefs, the polyribosome.”*

Answers falling into the third category, which referred only to individual traits, accounted for 11 out of 66 answers. The third category was assigned when it was unclear whether newly learned concepts were incorporated into the answer. For example, student 51, who identified with the Golgi apparatus, wrote: “*I like organizing things like to-do lists, schedules, and my room. But after the course I also think that I like to help people and without the Golgi apparatus helping transport of molecules, the cell would not function.*” [sic].

To earn the bonus, the students were specifically instructed to incorporate newly learned material into their answers. In 11 cases, mostly from category 3, students did not receive the bonus point.

### Qualitative analysis

Table 1 invites further analysis of the types of personality traits and individual life experiences reflected in the responses. Several recurring themes could be identified from the data.

#### Personality traits and personal growth

An undergraduate college is a place for personal development and growth. Eight students (2, 4, 6, 9, 13, 49, 60, and 63 in Table 1) made references to personal growth. Two of them (2 and 13) linked the double-layered membranes of mitochondria to the multiple layers of one’s personality. As student 2 described it: “*Because I also have two layers (aka personalities). One that a lot of people see (outer membrane is permeable for* var*ious small molecules) and one that only certain people can see (those who pay for tickets in the theater.”* Another student who identified with mitochondria (student 6) wrote*: “I bring an uplifting energy to any room I’m in.”*

#### Organizational skills

Apparently, cell structures serve as metaphors for organizational skills, especially within the context of AUC’s educational policy of continuous assessment. This is an important theme for honors college students, who take 4 courses that require 20 assignments in a single semester. Cells function as organizers, and this was recognized in no fewer than 10 student responses (1, 15, 18, 24, 34, 48, 51, and 64). Examples include the following: Student 24, on the cytoskeleton, wrote: *“They are the real supporters and organizers of a cell. And I believe my best trait is my organizational skill.”* Student 15, on the mitochondria, wrote*: “… In many ways, the mitochondria is an organelle that aspire to be me: an intricate system which although overly complicated, manages to do things efficiently. I can at times rush into projects, but I always try to reach smaller goals one at a time and believe in the importance of taking your time and going step by step. Clearly it is working for the mitochondria.” [sic]* Student 34, on ribosomes, wrote*: “I identified as a ribosome because I am generally the type of person who likes ensuring everything organized and functions smoothly and efficiently.”* Student 51, inspired by the Golgi apparatus, wrote*: “I like organizing things like to-do lists, schedules, and my room. But after the course I also think that I like to help people and without the Golgi apparatus helping transport of molecules, the cell would not function.” [sic].*

#### Friendships and family ties

New friendships and sustaining strong family ties are important for undergraduate students. Six students (11, 21, 39, 50, 52, and 65) referred to this theme. The cytoskeleton was mentioned by one of the students (21): “*It provides support to the cell (my community) and helps transport molecules (I like helping and interacting with others).”* A student who identified with the cell membrane (39) phrased it as follows: “*I connect well with others as the proteins (integrins) in the membrane can connect with its surroundings and desmosomes and intermediate filaments can connect adjacent cells.” [sic].*

#### Physical activity

Sports play an important role in many students’ lives. Cells are capable of movement—such as hematopoietic cells—and possess the necessary machinery to move efficiently. In addition to the many students who referred to mitochondria, three students (22, 33, and 48) noted this theme in connection with other cell structures. The microtubular system is specialized in the movement of organelles within cells. A student (48) who chose microtubules eloquently combined cell biological knowledge with personal reflection: *“They are the “railroad” of the cell and help stuff move around, which I feel like I do every day. They also act like the logistic manager and move in the cell, which again, is similar to me.”*

#### Hobbies

In addition to physical activity and sports, other hobbies such as cleaning and cooking/ gastronomy were mentioned by students 35, 42, 44, 45, and 62. Cooking was associated with lysosomes—the organelles that digest or degrade biomolecules in the cell—by three students. Student 44 wrote: *“It can form a phagolysosome to degrade the proteins of the pathogen. Since I digest food easily, I resemble it.”*

#### Adaptation

The ability of cells to respond to new environmental clues had been discussed in the class on many occasions. Two students (16 and 27) referred to adaptation in the context of their chosen cell compartment. Student 16 commented on mitochondria as follows: *“I am adaptable, just like how mitochondria have antioxidant enzymes to protect against oxidative stress from ROS.”*

#### A sense of humor

While several responses were met with amusement, four in particular (responses 20, 32, 36, and 57) stood out due to their notably humorous content.

Student 20, on mitochondria, wrote: *“It metabolizes fatty acids and I’m excited to eat lots of fatty foods on Christmas day.”*

Student 32, on cytoplasm, wrote: *“Is the land of opportunities. Where proteins are synthesized and dreams are made.”*

Student 36 combined knowledge of ribosomes with mutations and mistakes*: “I am not a native speaker of English, and I need to translate information and sometimes also make mistakes (mutations).”*

Finally, student 57, on the GPCR receptor, wrote: *“The GPCR receptor passes the membrane 7 times and has 3 subunits. My birthday is on 7th of March, so I identify with it.”*

## Discussion

The answers to the final exam question in a Molecular Cell Biology course, in which students were asked to choose a cellular component they had learned about, provided substantial insights. A major finding was that many students were able to articulate the new concepts and knowledge they had gained during the course. Although cellular components such as the cytoskeleton were not mentioned during the elevator pitch on the first day of the course, they appeared in the final exam responses, demonstrating that the students had gained a more comprehensive understanding of cellular components. No fewer than 21 cellular components were mentioned by the 66 students, with mitochondria being the most frequently cited.

Students were not only capable of providing purely scientific answers (20 out of 66) but were able to combine novel cell biological findings with their personal traits (35 out of 66). Finally, 11 students only mentioned personal traits, as they had done during the get-to-know activity. The high number (35 + 11) that either scored hybrid or purely on personal traits, was unexpected, given the nature of the question. Likely, the introduction of the question invited students for hybrid answers.

The types of answers the students gave regarding personal traits reflect the significant life changes associated with studying in college. How students cope with a new environment is clearly on their minds, as seen in their responses regarding both hybrid and personal trait. New friendships, planning, sports participation, and adaptation were mentioned frequently. Overall, college students often experience high and long-lasting stress levels (13). Finding a sense of belonging is important, since it may foster stability (14) during life on campus. Improving quality of life can help relieve stress (15), as can the development of organizational skills (16), another trait mentioned by the students. Many hybrid and personal trait responses were further related to sports. Encouraging the combination of sports and academic life as an undergraduate has been shown to positively correlate with study outcomes (17).

The study demonstrates how the diverse characteristics of cells can evoke a wide range of associations, reflecting the complexity of their own properties. Cells are linked to concepts such as motion, stability, double membranes, shifting protein behaviors, and mutations—features that also echo aspects of human personality.

## Data Availability

The original contributions presented in the study are included in the article/supplementary material, further inquiries can be directed to the corresponding author.
